# TWIST-MR-Angiography to aid central venous access in challenging patients. A single centre experience

**DOI:** 10.1186/1532-429X-18-S1-T8

**Published:** 2016-01-27

**Authors:** Chris B Lawton, Jonathan C Rodrigues, Lynne Armstrong, Nathan E Manghat

**Affiliations:** CARDIAC MRI UNIT, Bristol Heart Institute, Bristol, United Kingdom

## Background

Obtaining adequate venous access is a recurrent problem in patients where long term access is required for the intravenous administration of prolonged antibiotics or parenteral nutrition. Assessment of vein adequacy and patency is clinically important in these patients.

**Figure 1:**
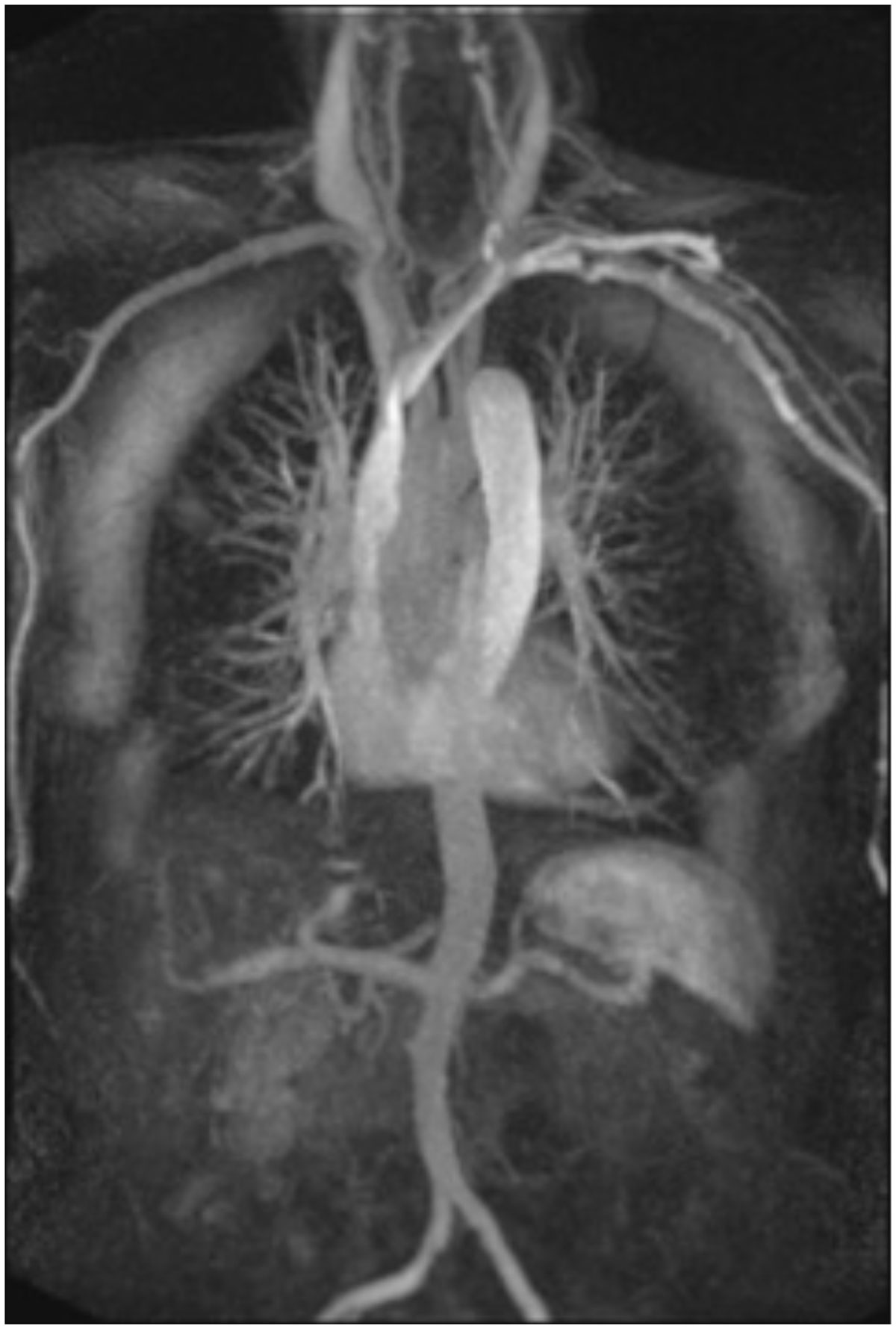
Figure 1

Time Resolved Angiography with interleaved stochastic trajectories (TWIST) is a technique that creates a sequential series of multiplanar images during passage of intravenous contrast (Figure 1). TWIST has proven to be very useful in the assessment of vascular haemodynamics such as in arteriovenous malformations (AVMs), fistulas and shunts as well as the investigation of central thoracic veins. Our centre routinely uses TWIST MRA in patients with complex congenital heart disease, particularly for the assessment of complex cardiac connections such as repaired Tetralogy of Fallot, and demonstration of the Fontan circulation.

## Methods

We retrospectively reviewed patients who successfully underwent TWIST MR angiography at our centre for the evaluation of central venous patency with a view to obtaining access. 70% of patients were on long term total parenteral nutrition (TPN) for intestinal failure, the remaining 30% were patients with cystic fibrosis requiring long term lines for antibiotic administration).

TWIST MRA in parallel with the GeneRalized Autocalibrating Partially Parallel Acquisitions (GRAPPA) algorithm was performed using a 1.5T Siemens Magnetom Avanto MRI scanner. Gadolinium contrast (dose of 0.1 mls per kg), was administered at a rate of 2 mls per second for each examination.

## Results

**Figure 2:**
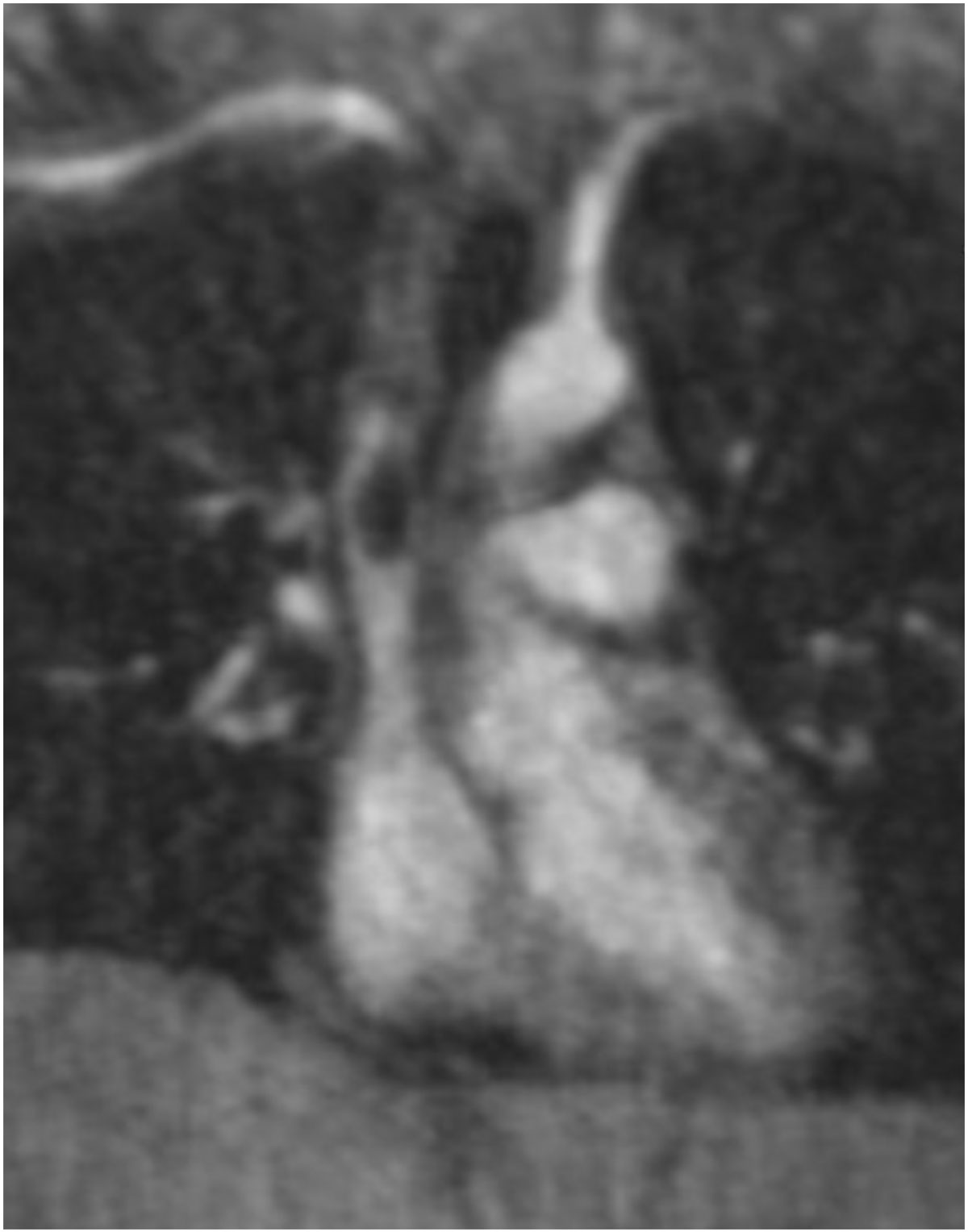
Figure 2

All studies were diagnostic and in all cases vascular occlusive disease was confirmed. In 84% of all cases an appropriate site for access was recommended, with successful implementation in 84%. Access was not attempted in the remaining patients. In one patient, the technique was performed to identify a suspected superior vena caval obstruction (Figure 2). One patient had previously undiagnosed congenital IVC interruption and there were several cases of internal jugular vein and subclavian vein occlusion.

## Conclusions

We believe that TWIST with GRAPPA parallel acquisition could be used successfully to non-invasively and efficiently image patients with more complex vascular access issues, including those requiring long term total parental nutrition (TPN).

